# Emotional Well-Being Animal Models

**DOI:** 10.1093/geroni/igab046.782

**Published:** 2021-12-17

**Authors:** Kuan Wang

**Affiliations:** University of Rochester, Rochester, New York, United States

## Abstract

Clinical studies suggest an association between EWB and the risk or progression of AD. However, the mechanistic link and causal relationship between EWB and AD remain unknown, due to limited experimental access and control of the underlying human brain processes. Animal models offer genetic control of AD mutations and neural circuit analysis tools, but subjective feelings of EWB cannot be assessed through self-report. To study EWB across species, we adopt a theoretical framework that views emotions as central brain states that respond to exteroceptive or interoceptive stimuli and cause multiple cognitive, somatic and behavioral changes. Recent neuroanatomical and functional imaging studies have identified evolutionarily related brain circuits in the encoding and regulation of central emotional states in animals. Dr. Wang will review progress in elucidating the functional activities of these circuits and discuss the challenges and opportunities to link these neural representations to EWB and AD related pathological progression.

